# Self-medication practices and the content of home pharmacies in the households of pharmacy and medical students in Zagreb, Croatia, in 2022 with a reference to the findings from 2001 and 1977

**DOI:** 10.3325/cmj.2025.66.247

**Published:** 2025-08

**Authors:** Vedrana Aljinović-Vučić, Ana-Marija Vargantolić, Davor Virag, Marin Vučić, Zdravko Lacković, Vladimir Trkulja

**Affiliations:** 1 Department of Basic and Clinical Pharmacology and Toxicology, Rijeka University School of Medicine, Rijeka, Croatia; 2 Health Care Center Zagreb-Zapad, Zagreb, Croatia; 3 Department of Basic and Clinical Pharmacology, Zagreb University School of Medicine, Zagreb, Croatia; 4 Zagreb University School of Medicine, Zagreb, Croatia; 5 Department of Basic and Clinical Pharmacology, Zagreb University School of Medicine, Zagreb, Croatia (retired)

## Abstract

**Aim:**

To inventory the content of home pharmacies and evaluate drug keeping and self-medication practices in the households of medical and pharmacy students at Zagreb University in 2022, and to relate the findings to two previous surveys.

**Methods:**

A cross-sectional survey enrolled 178 students who inventoried drug supplies in their family households, and interviewed household members on drug keeping and self-medication practices. Previous surveys included 287 (in 2001) and 225 (in 1977) students/households.

**Results:**

In most households, medicines were kept at a designated place (around 65%, all surveys) but commonly contained products past the expiry dates or of unknown purpose (59% vs 27%-32% previously). Analgesics-antipyretics were kept in practically all households (93%-97% across all surveys), followed by cough-cold relief products (55% vs 30%-33% households) and anti-allergic drugs (systemic) (44% vs 25% households). These drug classes were commonly used for self-medication (all surveys). Of the typical prescription drugs, the most common were benzodiazepines (34% vs 24% previously), which were at least occasionally used for self-medication in 40% of such households. Systemic antibiotics were found in 17% of the households (vs 40%-46% previously), and the tendency of self-medication was less common (20% vs around 40% of the households).

**Conclusions:**

The patterns observed in households with expected above-average drug literacy and access (future health care professionals, health care professionals common among household members) in three surveys completed across 50 years were discrepant with “responsible self-medication” (drug handling, storage, usage). As such, they emphasize a general need for improved awareness about this topic.

The World Health Organization (WHO) defines “self-medication” as a practice that “…involves the use of medicinal products by the consumer to treat self-diagnosed disorders or symptoms, or the intermittent or continued use of medication prescribed by a physician for chronic or recurrent diseases or symptoms” (1). The “consumer” refers to lay persons not formally qualified for diagnosing medical conditions and the use of medicinal products (medications). The definition implies that: i) lay persons self-diagnose a condition and decide about the treatment completely without professional guidance; ii) lay persons recognize symptoms of a condition initially diagnosed by a physician but autonomously decide upon the use of a prescribed medication. However, self-medication includes a variety of other practices, such as self-initiated and self-managed use of medications that should be used exclusively if prescribed and supervised by a health care professional (prescription-only medication), completely unsupervised use of various “traditional”/herbal preparations, and unsupervised administration of medicines (of any kind) to family members and friends (2-4).

Formally, intended for self-medication are medicinal products that are to be used for treatment of conditions/symptoms readily recognizable by lay persons, and have been judged as effective and safe, ie, medicinal products that may be used – at least over some limited period of time – without medical supervision. As such, they are eligible for direct consumer advertising and can be obtained without a prescription (over the counter [OTC]) (2). In the past few decades (at least in Europe), a growing number of medicinal products have been reclassified regarding the mode of their supply from “prescription-only” to OTC products, thus becoming increasingly available to patients (2). In many cases, they have even become more affordable and therefore available to the general population (5,6). If “responsible” (or practiced as intended), self-medication may relieve the health care system of a considerable burden of managing minor ailments, and enables patients to independently decide about management of such conditions in line with individual preferences (5,7). On the other hand, self-medication can be a health threat and result in an increased burden to the system, as a consequence of incorrect treatment (missed diagnosis, use of inappropriate medicines, and/or doses or duration of treatment), abuse, potential for drug-drug interactions and polypharmacy, delay in needed treatment, increased morbidity, and in the most severe cases – even mortality, depending on which drugs are self-medicated and by whom (7-9). Even the most common and “benign” regularly OTC medicines, such as antipyretics, may be seriously harmful if not used properly (10). The paradigmatic example of inappropriate self-medication is the medically unsupervised use of antimicrobials, which considerably contributes to the increasing problem of antimicrobial resistance (11-15).

Based on the data from the third wave of the cross-sectional European Health Interview Surveys conducted across the European Union member states in 2018-2020, 34.3% (weighted average) of the non-institutionalized residents ≥15 years of age reported that they had practiced self-medication at least once over the preceding 14 days, with country-specific prevalence ranging from 15% to 70% (16). Considering such a high prevalence, and associated potential benefits as well as harms, self-medication has become an important topic in health care. In 2004, while preparing a manuscript on self-medication, we searched PubMed using the terms “self-medication” OR *“*auto-medication,” and retrieved 890 articles (17). An identical search in December 2024 retrieved 40 481 “hits” – rough but illustrative indication of a growing interest in this topic.

In 1977 (18), to increase the awareness of future health care professionals about the importance of adequate handling of medicines in daily life, and their appropriate use, we conducted a survey among third-year medical and pharmacy students at Zagreb University. At the start of their pharmacology courses, students were invited to inventory the drug supplies in their own family households, to conduct a structured interview with the household members, and to complete a questionnaire pertaining to the self-medication practices in their households. In 2001 (17), we repeated the survey using the same methodology, aiming primarily to assess possible changes or trends over a time period during which fundamental socio-economic changes had occurred (disintegration of the former state, transition to liberal democracy). In 2022, we conducted a third, identically conceived and executed survey. In the 20 years since the previous survey, Croatia had become a European Union member state fully accepting European medicines legislation and had experienced further development of the market economy and the pharmaceutical sector. We here report the results with a focus on trends of the main findings across the three surveys over a period of almost 50 years.

## Participants and methods

The anonymous survey was conducted during the academic year 2021/2022 at the Faculty of Pharmacy and Medical Biochemistry and the School of Medicine, Zagreb University, and was approved by the Ethics Committees of both institutions. All pharmacy students starting their course in drug metabolism, and all medical students starting their pharmacology course were invited to participate, received written information about the study objectives, together with the instructions on the type of information sought, and were instructed on how to inventory the household drug supplies and how to interview their household members. To enable comparability of the data with those collected in the previous surveys, the questionnaire and the mode of interview were the same as used previously (17,18). The questionnaire consisted of several multiple-choice questions to collect information on i) the school of attendance (either of the two); ii) whether there were health care professionals among the household members knowledgeable (expected by training) about adequate drug use (no/yes; medical doctors, doctors of dental or veterinary medicine, pharmacists); iii) the characteristics of keeping medicines within the household (no medicines in the household; designated secured place [“home pharmacy”]; no specific designated place [eg, scattered around the household]); iv) the predominant/main sources of information about the use of medicines (independent [not household members] health care professionals; health care professionals – household members; product information [leaflets; lay persons, eg, friends, family]; v) medicines kept within the reach of children (no medicines/children <14 years of age in the household; medicines kept out of or within the reach of children); vi) keeping medicines of unknown purpose or/and medicines past the expiry dates (neither are found; medicines of unknown use present, medicines past the expiry dates present; both); vii) keeping medicines that could exert toxic effects (based on product information leaflets). The collected inventories were subsequently reviewed by two medical doctors and a pharmacist; viii) the questionnaire also included a standardized spreadsheet to record the trade name and generic name for each medicine found; unit strength and pack size; number of packs; whether the purpose/indication was known; and practice of self-medication related to the specific product. Self-medication was defined as the use of drugs without prior consultation with the physician regarding the indication, dosage, and duration of treatment, and it included two possibilities: (a) the patient completely independently decides on the use of a medicine, and (b) a certain medicine was initially prescribed/recommended by a physician but during the course of treatment the patient changes its dosage or duration or stops the treatment and restarts it according to their own will or uses the drug to treat another disease. These questions were asked for each individual drug found in the household with three possible answers: “never,” “sometimes,” “regularly” (self-medicated). The participants were given time till the end of the course to conduct the survey and return the questionnaires. Once collected, the data were reviewed by a pharmacist and two medical doctors – individual compounds were pooled into pharmacodynamic or pharmacotherapeutic groups. The survey was descriptive in nature with no inference intended.

## Results

### Households and household drug supplies

Of the 378 invited students, 178 (47%) agreed to participate and returned generally valid questionnaires. In 22.0% of the surveyed households, at least one member was a health care professional knowledgeable about adequate use of medicines. In 62.9% of the households, drugs were kept at a designated place (Table 1). The supplies commonly contained medicines past the expiry dates (43.3%) (Table 1), and drugs past the expiry dates or of unknown purpose were found in 51.2% of the households (Table 1). Children younger than 14 years of age were reported in only 39 households, but for 33 of them (84.6%) drug supplies were within the reach of children (Table 1).

**Table 1:** General characteristics of drug supplies in the surveyed households (valid N = 178)

Characteristic	Number (%) of households
Drugs are kept:*	
at a designated place – “home pharmacy”	112 (62.9)
at several places around the house	68 (38.2)
there are no drugs in the household	2 (1.1)
not specified	4 (2.2)
Availability of drugs to children <14 years of age	
no children in the household	139 (78.1)
children in the household – drugs out of reach	6 (3.3 or 15.4^✝^)
children in the household – drugs within reach	33 (18.5 or 84.6^✝^)
Drug shelf-life and purpose	
there are drugs past the expiry dates	77 (43.3)
there are drugs of unknown purpose	14 (7.9)
there are drugs past the expiry dates and of unknown purpose	14 (7.9)

*Sums up to >178, since answers (i) and (ii) were not mutually exclusive.

✝If only households with children <14 years of age are counted (n = 39).

Analgesics-antipyretics for systemic use were found in 96.6% of households, followed by various cough-cold products (55.1%), non-steroidal topical preparations (45.5%), and anti-allergic drugs for systemic use (43.8%) (Table 2). A variety of other drug products belonging to different pharmacodynamics/pharmacotherapeutic classes were found in around 1/3 of the households (Table 2). Of note, benzodiazepines and systemic antibiotics-antimicrobial chemotherapeutics (typically not intended for self-medication) were found in 33.7% and 16.8% of the households, respectively (Table 2).

**Table 2:** Drug classes identified in the surveyed households (valid N = 178)

Pharmacodynamic or pharmacotherapeutic group	Number (%) of households
Analgesics-antipyretics, systemic	172 (96.6)
Cough-cold products (non-opioid antitussives, rhinologics, combinations with NSAIDs/first-generation antihistamines)	98 (55.1)
Topical non-steroidal products (solutions, semi-solids)	81 (45.5)
Anti-allergic drugs, systemic	78 (43.8)
Drugs for treatment of the peptic disease	64 (36.0)
Benzodiazepines	60 (33.7)
Antihypertensive drugs including diuretics	48 (27.0)
Antithrombotics (antiplatelets, sporadically anticoagulants)	39 (21.9)
Vitamins and nutrients	33 (18.5)
Topical steroid products	32 (18.0)
Opioid antitussives/analgesics/antidiarrheals (pholcodine, tramadol, loperamide)	31 (17.4)
Antibiotics-chemotherapeutics, systemic (overall)	30 (16.8)
beta-lactam antibiotics	20 (11.2)
other (including systemic antiviral and anti-fungal agents)	20 (11.2)
Spasmolytics	27 (15.2)
Oral contraceptives	17 (9.6)
Drugs to treat asthma and/or COPD	14 (7.9)
Hypolipemics	14 (7.9)
Hormones, systemic (except oral contraceptives)	12 (6.7)
Antidiabetics	10 (5.6)
Other (cumulatively)	83 (46.6)

*Abbreviations: COPD – chronic obstructive pulmonary disease; NSAID – non-steroidal anti-inflammatory drugs.

### Self-medication practices

Generally, in most of the households the use of medicines was judged as adequately informed, since the main sources of information were medical doctors or pharmacists (not family members) (58.4%), product information leaflets (18.0%), and household/family members who were health care professionals (9.6%) – cumulatively 153/178 (86%) (Table 3).

**Table 3:** The main sources of information about drug use (valid N = 178)*

	Number (%) of households
Independent^✝^ medical doctor and/or pharmacist	104 (58.4)
Product information leaflet	32 (18.0)
Household/family member who is a health care professional	17 (9.6)
Not a health care professional (eg, friends, relatives)	2 (1.1)
Various – could not consent about one preferred choice	23 (12.9)

*Members of the surveyed households were interviewed in respect to the main source of information about rational drug use. A consensus statement for each household about the predominant behavior was recorded.

✝Independent – not a household/family member.

Specific drug classes that were most commonly found in the household drug supplies (Table 2) were also typically used for self-medication although not exclusively as a regular practice (Table 4). By drug classes, the proportion of households in which self-medication was practiced at least occasionally (“sometimes”) was as follows: i) systemic analgesics-antipyretics in 95% of the households, ii) cough-cold relief products in 84%, iii) topical non-steroidal products in 82%, and iv) anti-allergic products in 68% (Table 4). These are the drug classes largely intended for self-medication, since used for regularly recognizable minor ailments. Systemic antibiotics/antimicrobial agents – prototypical compounds that should not be used for self-medication – were found in only 30 households, and for 23/30 (77%) self-medication was explicitly excluded (“never”) (Table 4). On the other hand, benzodiazepines – psychoactive substances that are also generally not intended for self-medication – were found in 60 households, and at least occasional (“sometimes”) self-medication was reported in 24 of them (40%) (Table 4).

**Table 4:** Prevalence (n [%])of self-medication practices of specific pharmacodynamic/pharmacotherapeutic drug classes in households in which they were found

Drugs (pharmacodynamic pharmacotherapeutic group)	N of households	Never	Sometimes	Regularly	Not specified
Analgesics-antipyretics, systemic	172	7 (4)	144 (84)	19 (11)	2 (1)
Cough-cold relief products (non-opioid antitussives, rhinologics, combinations with NSAIDs/1st generation antihistamines)	98	14 (14)	75 (77)	7 (7)	2 (2)
Topical non-steroidal products (solutions, semi-solids)	81	14 (17)	53 (65)	14 (17)	0
Anti-allergic drugs, systemic	78	23 (29)	41 (53)	12 (15)	2 (3)
Drugs for treatment of the peptic disease	64	27 (42)	25 (39)	9 (14)	3 (5)
Benzodiazepines	60	31 (52)	20 (33)	4 (7)	5 (8)
Antihypertensive drugs including diuretics	48	26 (54)	1 (2)	18 (38)	3 (6)
Antithrombotics (antiplatelets, sporadically anticoagulants)	39	17 (44)	14 (36)	4 (10)	4 (10)
Vitamins and nutrients	33	15 (46)	7 (21)	9 (27)	2 (6)
Topical steroid products	32	14 (44)	17 (53)	1 (3)	0
Opioid antitussives/analgesics/antidiarrheals (pholcodine, tramadol, loperamide)	31	21 (68)	7 (23)	1 (3)	2 (6)
Antibiotics-chemotherapeutics, systemic (overall)	30	23 (77)	5 (17)	1 (3)	1 (3)
beta-lactams	20	15 (75)	5 (25)	0	0
other (including systemic antiviral and anti-fungal agents)	20	14 (70)	3 (15)	2 (10)	1 (5)
Spasmolytics	27	16 (59)	10 (37)	0	1 (4)
Oral contraceptives	17	5 (29)	0	11 (65)	1 (6)
Drugs for asthma and/or COPD	14	8 (57)	0	4 (29)	2 (14)
Hypolipemics	14	5 (36)	9 (64)	0	0
Hormones, systemic (except oral contraceptives)	12	8 (66)	0	2 (17)	2 (17)
Antidiabetics	10	7 (70)	0	3 (30)	0
Other (cumulatively)	83	36 (43)	26 (31)	19 (23)	2 (3)

*Abbreviations: COPD – chronic obstructive pulmonary disease; NSAID – non-steroidal anti-inflammatory drugs

### Reference to previous surveys

The findings of the present study are similar to the main findings of our previous surveys in several aspects (Table 5): i) in most households, drug supplies were kept at a designated place (eg, “home pharmacy”) (around 65%); ii) in most of the households, the mode of use of medicinal products (around 95%) may be considered “adequately informed;” iii) systemic analgesics-antipyretics were a mandatory constituent of the household drug supplies (around 95%), and were typically used for self- medication (around 90%-95%); iv) various systemic or topical cough-cold relief products were regular contents of the household supplies (around 35%-55%), and were commonly used for self- medication (around 67%-84%); v) systemic anti-allergic products were also commonly present (around 25%-40%), and used for self- medication; vi) benzodiazepines were present in around 25%-35% of the households, and also commonly used for self- medication (around 50% of the households). On the other hand, compared with the previous surveys, in the present one we observed (Table 5) the following: i) a higher proportion of households keeping drugs past the expiry date or of unknown purpose (around 60% vs around 30%), ii) a higher proportion of households with children <14 years of age in which drugs were kept within the reach of children (around 85% vs around 25%-35% in previous surveys). However, the absolute number of households with children <14 years of age was low in all three surveys – 21.9% (n = 39) in the present survey, 20.6% (n = 59) in 2001 (17), and 22.2% (n = 50) in 1977 (18); iii) a lower proportion of households keeping drugs (type, amount) that may cause severe toxicity (around 20% vs around 40%); iv) fewer households keeping systemic antimicrobials (around 17% vs around 45%), and v) fewer households with at least occasional self-medication of systemic antimicrobials (around 20% vs around 40%).

**Table 5:** The major findings of the current and our previous surveys (in 2001 and 1977): data are expressed as percentages of households for which a particular feature was recorded. Outcomes showing apparent trends across the surveys are bolded

	2022 (current)	2001 (17)	1977 (18)
Number of surveyed households	178	287	225
Healthcare professionals (HCP) in the household	22.0	36.9	32.9
Drugs kept at a designated place	63.3	68.3	64.9
Drugs past the expiry date or unknown purpose or both	**59.3**	**26.5**	**32.0**
Drugs (by type/amount) might cause severe toxicity	**20.3**	**44.3**	**43.1**
Household with children <14 years of age	21.9	20.6	22.2
In households with children, drugs within reach	**84.6**	**36.2**	**26.6**
The use of drugs is informed (by HCPs/product inserts)	98.7	92.3	95.6
Analgesics-antipyretics (systemic) found in home supplies	96.6	96.7	93.3
Where found, analgesics-antipyretics self-medicated	95.0	87.9	95.1
regularly	11.0	28.0	NR*
sometimes	84.0	59.9	NR
Cough-cold relief products found in home supplies	55.1	33.1	30.0
Where found, cough-cold relief products self-medicated	84.0	67.0	NR
regularly	7.0	15.0	NR
sometimes	77.0	52.0	
Anti-allergic drugs (systemic) found in home supplies	43.8	25.2	NR
Where found, anti-allergic drugs self-medicated	68.0	50.0	NR
regularly	15.0	5.0	NR
sometimes	53.0	45.0	
Antibiotics (systemic) found in home supplies	**16.9**	**46.3**	**40.4**
Where found, antibiotics self-medicated	**20.3**	**37.7**	**41.3**
regularly	3.3	10.5	NR
sometimes	17.0	27.2	NR
Benzodiazepines found in home supplies	33.7	23.7	NR
Where found, benzodiazepines self-medicated	40.0	57.0	NR
regularly	7.0	9.0	NR
sometimes	33.0	48.0	NR

*NR – not recorded.

## Discussion

The main present and previous findings could be classified into three categories. The first group refers to “reasonable and expected findings”: i) analgesics-antipyretics for systemic use were found in practically all households in all surveys; ii) various cough-cold relief products (systemic or topical) and systemic anti-allergic drugs were also commonly found, in somewhat more households in the present than in the previous surveys. This might be due to the fact that over the past 20 years many such products have been granted the OTC status; iii) regarding these three drug classes, self-medication consistently appeared a common practice, which is expected since they are intended for regularly recognizable minor ailments. The second group of findings could be denoted as “reassuring” with respect to responsible self-medication or keeping and use of drugs in general: i) across the surveys, virtually all households were judged as those in which the use of drugs was predominantly based on adequate information provided by health care professionals or product inserts; ii) a thorough review of all individually listed drug products in each household indicated that the proportion of households in which drugs (by type, unit strengths, and total amount) with a potential of causing severe toxicities was lower in the present (around 20%) than in the previous surveys (around 43%); iii) ideally, antibiotics should never be self-medicated for the well-known reasons (19). The proportion of households in which antibiotics for systemic use were found was considerably lower in the present (17%) than in the previous surveys (around 45%), as was the proportion of those in which at least occasional self-medication of antibiotics was practiced. Finally, we consider some of the observations to be “potentially worrisome”: i) benzodiazepines were found in 25% to 33% of the households and in around half of them, they were at least occasionally used for self-medication; ii) in around 66% of the households in all surveys, drug supplies were kept at a designated place, but commonly contained drugs past the expiry dates or of unknown purpose, and were commonly within the reach of children. The latter practice was more common in the current than in the previous surveys, although the number of households with children <14 years of age was modest in all surveys. We believe that the primary relevance of these observations is related to the risk of unintentional intoxication in children. Such events are rare (20). In a one-year observational global study (105 hospitals across all WHO regions) of pediatric intoxications, of the 363 245 recorded pediatric emergency department visits, only 494 cases (0.13%) were due to unintentional ingestion of medicinal products (20). However, (unintentional) intoxication with medicinal products was the most common cause of pediatric intoxication – 28.6% of all intoxications (494/1727) were of this type, as well as 42.7% (494/1157) of all unintentional intoxications (20). The most common individual culprits in these cases were benzodiazepines (14.0%), other psychotropics (7.7%), paracetamol (10.5%), and NSAIDs (7.7%) (20). Typically, the parents admitted that the drugs were not kept out of the reach of children (20).

Although the definition of self-medication by the WHO (1) appears unambiguous, different meanings have been assigned to this concept (21). A recent systematic review of “definitions” of self-medication indicated considerable heterogeneity of meanings implied under the term (21): i) in most cases, the definition was based on the process of obtaining the drug (eg, OTC), non-participation of a health professional (eg, self-diagnosis, lay person’s individual choice of a drug and dose), the source of the medicinal product (eg, pharmacy, or “free sales”), and reasons (eg, minor ailments); ii) other included non-adherence to a prescription, re-use of stored drugs, and sharing and lending (OTC or prescription) medicines among lay persons. WHO considers self-medication as a part of a broader concept of “self-care,” ie, one of the activities intended to promote, improve, or restore health and well-being (22). This implies that practice should result in benefits, ie, that it is “responsible.” The statement of the World Medical Association about self-medication of medicinal products (221st Council Session in Berlin, Germany, 2022) (23) explicitly states that “responsible self-medication” implies several dimensions of responsibility: i) it refers only to medicines approved for OTC distribution, which means that a positive risk-benefit assessment holds under the conditions of use managed by the lay persons; ii) people who choose to practice self-medication should be able to recognize the condition they are treating as one suitable for self-medication, to choose an appropriate product and mode of use, that is, to follow the direction for use provided in the product labeling; iii) health professionals must educate/advise patients about all the aspects of self-medication and instruct them to seek medical advice if unsure; iv) governments and regulators should ensure that individuals who self-medicate are well informed, and reinforce pharmacovigilance for self-medication; v) manufacturers are obliged to follow regulatory codes, provide full and understandable information about the product, and be responsible in advertising to public and marketing of the OTC medicines; vi) all parties involved should treat medicines (prescription and OTC) as special products by ensuring their safe distribution, usage, and storage. If perceived within the broader context of self-care, as, eg, implied by the WHO (22), then storage of medicines by the end-users might be viewed as an “extension” of the concept of self-medication in that it might reflect on the safety aspects: even if only OTC medicines are kept, there is reasonable ground for safety concerns if the amounts are large, or within the reach of children, and/or awareness of their purpose and mode of use is lacking. Adequate information on storage and eventual disposal of (unused) medicines is also a part of responsible handling of medicinal products, including those intended for self-medication (5). This might be of particular interest in exceptional circumstances such as during the COVID-19 pandemic, when limited access to direct contact with health care professionals and fear of the disease resulted in excessive purchases and stocking up of medicines (24,25).

The present and our previous surveys in 1977 (18) and 2001 (17) addressed a few general points about drug keeping and self-medication practices in the households of medicine and pharmacy students at Zagreb University. None of the samples was representative of any specific target population; hence, the observations should not be projected to, eg, “general Croatian population” or “population of health-science students,” and not even to the population of “medical and pharmacy students” (country/city-specific self-selection sample) – and this was never the intention. The initial survey in 1977 (18) was motivated by both research and educational purposes, and the 2001 (17) and the present surveys were motivated primarily by the intention to observe potential trends over time since the social paradigms substantially changed between the surveys: between 1977 (18) and 2001 (17) Croatia became an independent country after a four-year war, with fundamental changes in its socio-economic structure, whereas between 2001 and 2022, it became an EU member state and embraced EU medicines legislation. In all three surveys, we addressed the “same pool” of potential participants, ie, third-year medical and pharmacy students in Zagreb, with similar response rates (47% in the current survey, 68% in 2001, and 53% in 1977), using the same questionnaire to enable comparisons across the surveys. In essence, although we employed no formal “statistical tests” (as they would be meaningless in such a context), we tested a theory – and non-representative samples are a feasible choice for such a purpose (26). The fact that the used questionnaire was not validated, like some of the instruments described in the literature (27), would likely be a major limitation had the intention been to “reliably scan” the target population – which it was not. However, the present study contained elements not included in a variety of reported instruments in similar investigations. Hence, we considered that simple cross-tabulation of findings across the three surveys provided valuable information.

In the present survey, the inventory of home pharmacies found drugs falling into >15 different pharmacotherapeutic/pharmacodynamic classes, and a number of these – typical prescription drugs (eg, antihypertensives, antiplatelets, opioid antitussives/analgesics, drugs to treat asthma and/or chronic pulmonary obstructive disease) – were “at least sometimes” used for self-medication. Such practices may have detrimental health consequences – adverse effects or lack of therapeutic effect. However, the number of households in which these compounds were found was low, and we did not have a possibility to address possible trends over time or to reasonably draw any conclusions.

To sum up, the present and previous surveys, conducted across almost 50 years included participants/households for which it is reasonable to assume an above-average drug literacy and access to drugs (future health care professionals, health care professionals common among household members), and yet data indicated consistent discrepancies from “responsible self-medication” in the broadest sense (ie, handling, storage, usage). While some points may be considered “expected” or “reassuring,” some elements of household drug keeping (drugs accessible to children; drugs past the expiry dates or of unknown purpose) and their usage (self-medication of typical prescription drugs) were consistently far from desirable. As such, data emphasize a general need for improving public awareness about adequate keeping and use of drugs.

